# Pleiotropic activities of succinate: The interplay between gut microbiota and cardiovascular diseases

**DOI:** 10.1002/imt2.124

**Published:** 2023-06-19

**Authors:** Jing Xu, Yicheng Yang, Xin Li, Shusi Ding, Lemin Zheng, Changming Xiong, Yuejin Yang

**Affiliations:** ^1^ Department of Cardiology, State Key Laboratory of Cardiovascular Disease, Fuwai Hospital, National Center for Cardiovascular Diseases Chinese Academy of Medical Sciences and Peking Union Medical College Beijing China; ^2^ Respiratory and Pulmonary Vascular Center, State Key Laboratory of Cardiovascular Disease, Fuwai Hospital, National Center for Cardiovascular Diseases Chinese Academy of Medical Sciences and Peking Union Medical College Beijing China; ^3^ China National Clinical Research Center for Neurological Diseases, Tiantan Hospital, Advanced Innovation Center for Human Brain Protection The Capital Medical University Beijing China; ^4^ The Institute of Cardiovascular Sciences and Institute of Systems Biomedicine, School of Basic Medical Sciences, Key Laboratory of Molecular Cardiovascular Sciences of Ministry of Education, NHC Key Laboratory of Cardiovascular Molecular Biology and Regulatory Peptides, Health Science Center Peking University Beijing China

**Keywords:** biosynthetic pathway, cardiovascular diseases, gut microbiota, mechanisms, succinate

## Abstract

Cardiovascular diseases (CVDs) continue to be a significant contributor to global mortality, imposing a substantial burden and emphasizing the urgent need for disease control to save lives and prevent disability. With advancements in technology and scientific research, novel mechanisms underlying CVDs have been uncovered, leading to the exploration of promising treatment targets aimed at reducing the global burden of the disease. One of the most intriguing findings is the relationship between CVDs and gut microbiota, challenging the traditional understanding of CVDs mechanisms and introducing the concept of the gut‐heart axis. The gut microbiota, through changes in microbial compositions and functions, plays a crucial role in influencing local and systemic effects on host physiology and disease development, with its metabolites acting as key regulators. In previous studies, we have emphasized the importance of specific metabolites such as betaine, putrescine, trimethylamine oxide, and *N,N,N*‐trimethyl‐5‐aminovaleric acid in the potential treatment of CVDs. Particularly noteworthy is the gut microbiota‐associated metabolite succinate, which has garnered significant attention due to its involvement in various pathophysiological pathways closely related to CVDs pathogenesis, including immunoinflammatory responses, oxidative stress, and energy metabolism. Furthermore, we have identified succinate as a potential biomarker, highlighting its therapeutic feasibility in managing aortic dissection and aneurysm. This review aims to comprehensively outline the characteristics of succinate, including its biosynthetic process, summarize the current evidence linking it to CVDs causation, and emphasize the host‐microbial crosstalk involved in modulating CVDs. The insights presented here offer a novel paradigm for future management and control of CVDs.

## Highlights

- Cardiovascular diseases (CVDs) continue to be a significant contributor to global mortality, imposing a substantial burden and emphasizing the urgent need for disease control to save lives and prevent disability.
- The gut microbiota, through changes in microbial compositions and functions, plays a crucial role in influencing local and systemic effects on host physiology and disease development, with its metabolites acting as key regulators.
- Gut microbiota‐associated metabolite succinate involves in various pathophysiological pathways closely related to CVDs pathogenesis, including immunoinflammatory responses, oxidative stress, and energy metabolism.
- The burgeoning evidence surrounding the role of succinate in the gut microbiome carries significant implications for our comprehension of host‐microbiome interactions and the development of new treatments for CVDs.

## Graphical abstract

Current studies have suggested the pleiotropic functions of succinate in vascular endothelial injury, vascular smooth muscle cell proliferation and migration, ischemia–reperfusion injury, macrophage polarization, aortic aneurysm and dissection, regulation of the renin‐angiotensin system, cardiomyocyte hypertrophy, etc.

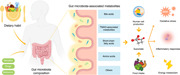

## INTRODUCTION

Cardiovascular diseases (CVDs) are the leading causes of mortality and disability, imposing a substantial medical and economic burden worldwide. According to the latest Global Burden of Disease 2019 Study, there were over 500 million diagnosed cases of CVDs, resulting in 18.6 million deaths in 2019 [1]. The increasing risk of CVDs has garnered significant attention, leading to a shift in management paradigms from treatment to prevention strategies. Despite extensive efforts in healthcare, CVDs continue to rise, particularly in middle‐ and low‐income countries, including China [1, 2, 3]. Hence, there is an urgent need for innovative approaches to assess and manage CVDs to achieve the targets set by the World Health Organization for Sustainable Development Goal 3 [4].

In the past decade, the study of human gut microbiota has emerged as a rapidly evolving research field, capturing the sustained interest of microbiologists, biological scientists, and clinicians [5]. The gastrointestinal system of the host is inhabited by a vast community of microorganisms, consisting of approximately 100 trillion microbes, with bacteria, archaea, fungi, and viruses being the predominant members [6, 7]. These microorganisms exist in a mutually beneficial relationship with the host and participate in various physiological interactions, such as nutrient absorption, digestion, fat metabolism, energy provision, and immunomodulation. These interactions collectively contribute to the maintenance of host homeostasis [8]. Thanks to the progress in high‐throughput sequencing and metagenomics in the early 21st century, the enigma surrounding human microbial communities has been gradually unraveled [9]. Consequently, the investigation into the role of gut microbiota in disease development, particularly in the context of CVDs, has become a prominent area of research. To date, a growing body of evidence has indicated the involvement of gut microbial variations in the pathogenesis of CVDs [10]. In comparison to healthy individuals, a significant decrease in the richness and diversity of gut microbiota was observed in hypertensive individuals. Furthermore, the hypertensive population was characterized by the identification of a gut enterotype predominantly composed of Prevotella [11]. Moreover, the gut microbiota also plays a crucial role in the development of heart failure [12] and pulmonary hypertension [13]. Atherosclerosis, a prevalent CVD, has been extensively studied for its association with gut microbiota. Multiple studies have provided substantial evidence linking atherosclerotic CVDs to elevated abundances of *Enterobacteriaceae* and *Streptococcus* spp. Abundances [14, 15]. The occurrence of atherosclerosis is influenced by molecular patterns derived from the gut microbiota [16]. Toll‐like receptors, a type of pattern recognition receptors, play a role in atherogenesis, and the commensal gut microbiota has been identified as a key activating factor for these pattern recognition receptors [17]. Microbial‐associated molecular patterns have been identified as significant contributors to the development of atherosclerosis. The utilization of germ‐free mouse models of atherosclerosis has allowed researchers to establish a causal relationship between gut microbiota and atherosclerosis. These models have aided in investigating the impact of gut microbiota on atherothrombosis [18], understanding the effects of microbiota on the size and cellular composition of atherosclerotic plaques [19], and analyzing how the interaction between gut microbiota and diet influences the progression of atherosclerosis [20].

**Figure 1:**
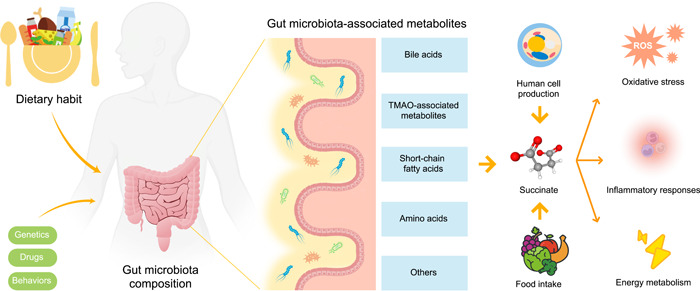
Gut microbiota‐associated metabolites. Gut microbiota contributes to the production of bioactive compounds, including short‐chain fatty acids, bile acids, amino acids, and TMAO‐associated metabolites such as TMAO, trimethyllysine, and *N,N,N*‐trimethyl‐5‐aminovaleric acid. Post dietary consumption, gut microbiota aids in generating metabolite precursors. Once these precursors enter the circulation, hepatic enzymes facilitate further metabolism, ultimately leading to the release of metabolites such as bile acid and TMAO into the circulatory system. Succinate is produced by certain gut bacteria as a metabolic byproduct. The presence of dietary fiber in the diet promotes the growth and activity of these bacteria, which in turn leads to increased production of short‐chain fatty acids (SCFAs), including succinate. Succinate plays a crucial role in oxidative stress, inflammatory responses, energy metabolism and other cellular processes. ROS, reactive oxygen species; TMAO, trimethylamine N‐oxide.

As a consequence of changes in the composition and function of gut microorganisms, the metabolites produced by the gut microbiota play a crucial role in regulating both local and systemic effects on host physiology and disease pathogenesis [21, 22]. The gut microbiota plays a role in the biosynthesis of various bioactive compounds, including bile acids [23], short‐chain fatty acids [24], amino acids [25], and trimethylamine/trimethylamine N‐oxide (TMAO) [26] (Figure 1). Importantly, the involvement of gut microbiota‐derived metabolites in CVDs has been extensively investigated [8, 27], underscoring their potential as therapeutic targets for CVDs intervention. In our previous research, we have demonstrated the effects of putrescine [28] and various metabolites in the TMAO pathway, including TMAO [29, 30, 31], betaine [32], and *N,N,N*‐trimethyl‐5‐aminovaleric acid [33, 34], on CVDs.

In recent times, there has been growing attention toward investigating the role of succinate, a gut microbiota‐associated metabolite, due to its involvement in various pathophysiological pathways closely associated with the pathogenesis of CVDs including hypertension [35], atherosclerosis [36], and cardiomyocyte hypertrophy [37]. Furthermore, we have identified succinate as a potential biomarker and demonstrated its therapeutic feasibility in the treatment of aortic aneurysm and dissection (AAD) [38]. In this review, we provide a comprehensive outline of the characteristics of succinate, summarize the existing evidence regarding its involvement in the development of CVDs, and emphasize the interplay between the host and gut microbiota in modulating CVDs. The valuable role of succinate in the metabolic interaction between the host and gut microbiota, along with extensive information on its relevance to CVDs, is thoroughly elucidated in this review. These insights pave the way for a novel paradigm in the treatment and management of CVDs, ultimately contributing to the reduction of the significant burden imposed by these diseases.

## SUCCINATE BIOSYNTHESIS BY HUMAN CELLS AND GUT MICROBIOTA

**Figure 2:**
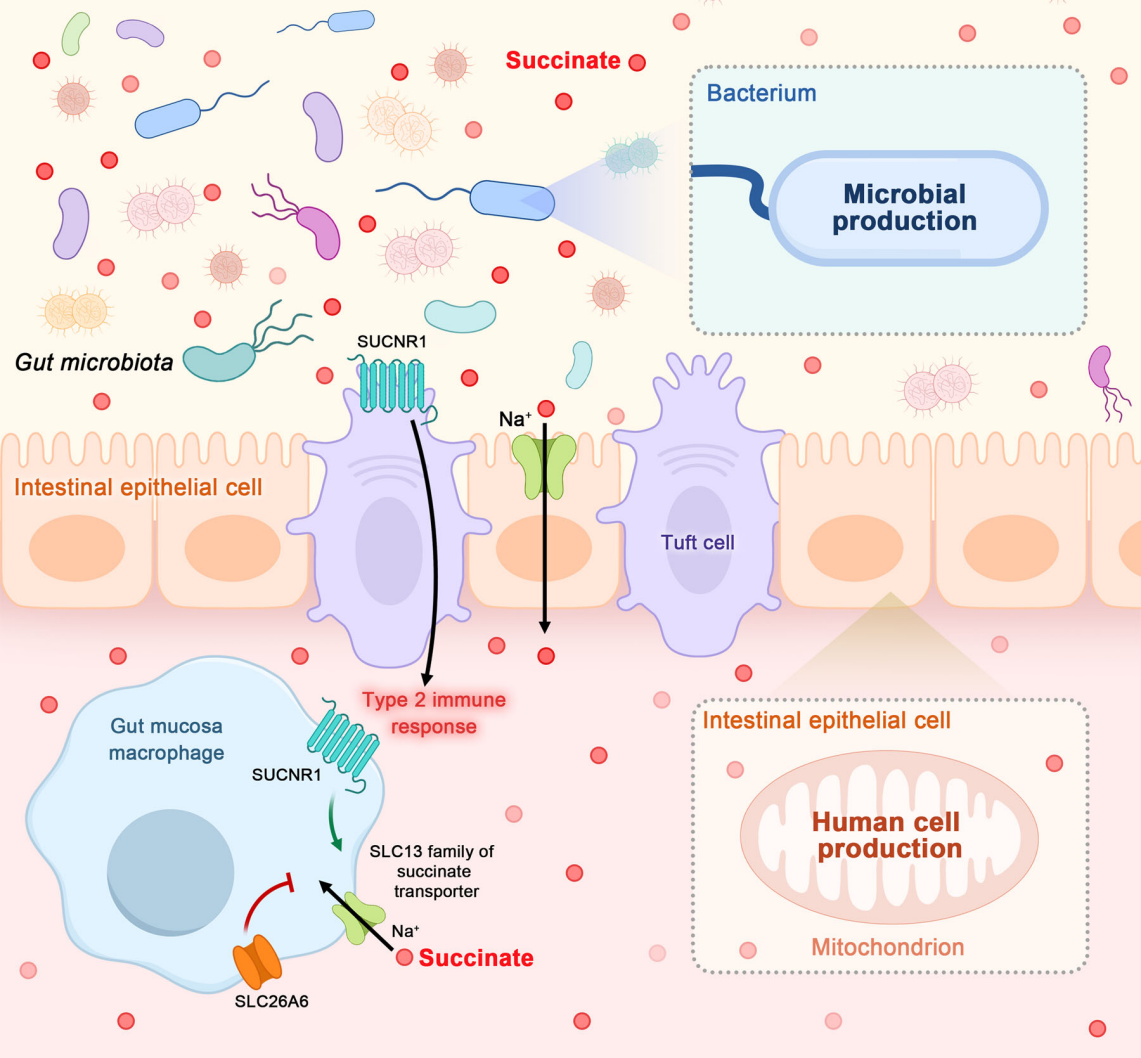
Succinate production and transportation in the human body. Succinate is synthesized by both human cells and gut microbiota. The charged nature of succinate enables its transport across plasma membranes, facilitated by the SLC13 family of Na^+^‐dependent transport proteins. Notably, the potent suppression of SLC13A2 occurs through the interaction with the SLC26A6 transporter. Furthermore, succinate plays a crucial role in extracellular signaling by stimulating the G protein‐coupled succinate receptor (SUCNR1), which is abundant in various tissues and cells. The uptake of succinate by macrophages can enhance and perpetuate inflammation, while its detection by tuft cells in the small intestine initiates type 2 immunity.

**Figure 3:**
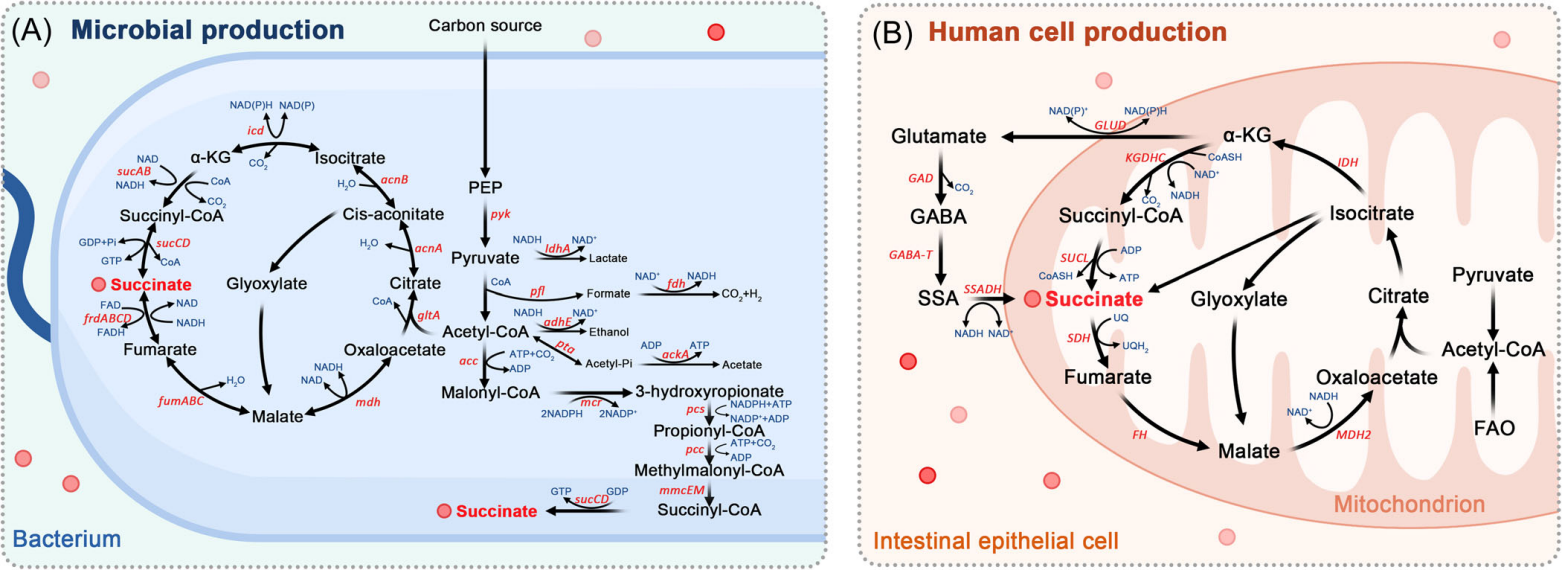
Biosynthetic pathways of succinate production in human cells and gut microbiota. (A) Succinate is commonly generated through a partial branch of the TCA cycle in microbial carbohydrate fermentation. The TCA cycle is present in nearly all microorganisms. Additionally, succinate can be produced through the glyoxylate shunt pathway and the 3‐hydroxypropionate pathway. (B) Succinate is an essential intermediate in the TCA cycle, which occurs in the mitochondria of host cells through a series of enzyme‐mediated reactions. In cells relying on anaerobic glycolysis or experiencing hypoxic conditions, alternative metabolic pathways are activated, leading to the accumulation of mitochondrial succinate. These pathways include the reductive branch of the TCA cycle through reverse succinate dehydrogenase activity, the GABA shunt, and glutamine‐dependent anaplerosis. *acc*, acetyl‐CoA carboxylase; *ackA*, acetate kinase; *acnAB*, aconitase; *adhE*, alcohol dehydrogenase; FAO, fatty acid oxidation; *fdh*, formate dehydrogenase; *FH*, fumarate hydratase; *frdABCD*, succinate dehydrogenase; *fumABC*, fumarate hydratase; GABA, γ‐aminobutyric acid; *GABA‐T*, GABA transaminase; *GAD*, glutamate decarboxylase; *gltA*, citrate synthetase; *GLUD*, glutamate dehydrogenase; *icd*, isocitrate dehydrogenase; *IDH*, Isocitrate dehydrogenase; *ldhA*, lactic dehydrogenase; *KGDHC*, ketoglutarate dehydrogenase complex; *mcr*, malonyl‐CoA reductase; *MDH2*, malate dehydrogenase isoform 2 (mitochondrial); *mdh*, malate dehydrogenase; *mmcEM*, methylmalonyl‐CoA epimerase and mutase; PEP, Phosphoenolpyruvic acid; *pcc*, propionyl‐CoA carboxylase; *pcs*, propionyl‐CoA synthase; *pfl*, pyruvate formate lyase; *pta*, phosphotransacetylase; *pyk*, pyruvate kinase; *SDH*, succinate dehydrogenase; *SUCL*, succinate‐CoA ligase; *sucABCD*, succinyl‐CoA synthetase; SSA, succinate semialdehyde; *SSADH*, succinate semialdehyde dehydrogenase; UQ, Ubiquinone; UQH2, Ubiquinol; α‐KG, alpha‐ketoglutarate.

Succinate, a C4‐dicarboxylic acid, is synthesized by both human cells and gut microbiota (Figure 2). Figure 3 illustrates the various pathways involved in the production of succinate.

## BIOSYNTHETIC PATHWAY IN HUMAN CELLS

The tricarboxylic acid (TCA) cycle, which serves as the eventual metabolic pathway for amino acids, sugars, and lipids, plays a crucial role in body metabolism. Within host cells, succinate is produced in mitochondria and serves as a substrate for mitochondrial oxidative phosphorylation. In the presence of an aerobic environment, pyruvic acid undergoes oxidative decarboxylation catalyzed by the pyruvate dehydrogenase complex, resulting in the production of acetyl coenzyme A (CoA). Subsequent adequate oxidation of acetyl CoA in mitochondria leads to the biosynthesis of succinate, accompanied by the liberation of energy. Under the function of succinate dehydrogenase, succinate is further dehydrogenated and metabolized to fumarate. Conversely, in cells relying on aerobic glycolysis or under hypoxic conditions, alternative metabolic pathways, including the gamma‐aminobutyric acid (GABA) shunt, the reductive branch of the TCA cycle involving reverse succinate dehydrogenase activity, and glutamine‐dependent anaplerosis, are activated. These pathways result in the accumulation of succinate in mitochondria [39].

## BIOSYNTHETIC PATHWAY IN GUT MICROBIOTA

In addition to being produced through the TCA cycle in human cells, succinate is also considered a bacterial metabolite found in the intestinal lumen and feces. Studies have demonstrated that germ‐free mice exhibit no detectable levels of succinate in their feces, highlighting the significant role of gut microorganisms in succinate biosynthesis [40]. Within microbial carbohydrate fermentation, succinate is predominantly generated via the partial branch of the TCA cycle, which is present in nearly all microbes. Similar to its production in human cells, the reductive branch of the TCA cycle, which converts oxaloacetate to succinate, serves as a pathway for succinate formation under anaerobic conditions [41]. However, unlike human cells, the glyoxylate shunt pathway is also considered as a vital biosynthetic route for succinate in gut microbiota [42]. In summary, acetyl‐CoA is converted to succinate, and under anoxic conditions, the glyoxylate shunt pathway becomes more active, leading to increased succinate production [41].

The identification of specific gut microbiota involved in succinate production and consumption provides valuable insights into the effects of this metabolite on disease physiology and pathogenic mechanisms. Notably, *Bacteroidaceae*, *Parabacteroides*, *Veillonella*, and Prevotella, including *Paraprevotella xylaniphila*, *Paraprevotella clara*, and *Prevotella ruminicola*, are among the major succinate producers [41, 43]. Bacteroides, the most prevalent members of the human flora, are anaerobic, gram‐negative bacteria with a rod shape and do not form spores. They coexist with humans and contribute to food digestion, energy production, and nutrient supply, including carbohydrate fermentation, utilization of nitrogenous substances, as well as bile acid and steroid bioconversion [44, 45]. Many gut bacterial strains possess glycolytic capabilities, enabling them to derive energy and carbon through the hydrolysis of carbohydrates. Succinate, acetic acid, and isovaleric acid are major byproducts of their anaerobic respiration. Additionally, certain *Ruminococcus* strains, such as *Ruminococcus flavefaciens* and *Ruminococcus albus*, also contribute to succinate biosynthesis. Conversely, succinate consumers include *Odoribacterium*, *Clostridium*, and *Phascolarctobacterium, including Phascolarctobacterium succinatutens* [46]. Table 1 provides an overview of the relative intestinal microbiota involved in succinate biosynthesis or metabolism.

**Table 1:** Summary of important gut microbiota producing or consuming succinate.

Phylum	Family	Genus	Species	Reference
Producing succinate				
Bacteroides	*Bacteroidaceae*	*Bacteroides*	*Bacteroides vulgatus*	[47]
			*Bacteroides fragilis*	[48]
			*B. thetaiotaomicron*	[49]
	*Prevotellaceae*	*Prevotella*	*Paraprevotella clara*	[50]
			*Paraprevotella xylaniphila*	[50, 51, 50, 51]
			*Prevotella ruminicola*	[52]
			*Prevotella copri*	[43, 46, 43, 46]
	*Tannerellaceae*	*Parabacteroides*	*Parabacteroides distasonis*	[53]
Firmicutes	*Ruminococcaceae*	*Ruminococcus*	*Ruminococcus flavefaciens*	[39]
			*Ruminococcus albus*	[48]
	*Lachnospiraceae*	*Blautia*	*Blautia wexlerae*	[47]
	*Ruminococcaceae*	*Desulfovibrio*	*Faecalibacterium prausnitzii*	[54]
	*Lactobacillaceae*	*Lactobacillus*	*Lactobacillus plantarum*	[55]
	*Veillonellaceae*	*Veillonella*	*Veillonella Parvula*	[46]
	*Selenomonadaceae*	*Mitsuokella*	*Mitsuokella multiacidus*	[49]
Actinobacteria	*Bifidobacteriaceae*	*Bifidobacterium*	*Bifidobacterium adolescentis*	[56]
			*Bifidobacterium animalis*	[56]
			*Bifidobacterium bifidum*	[56]
			*Bifidobacterium breve*	[56]
			*Bifidobacterium longum*	[56]
	*Propionibacteriaceae*	*Propionibacterium*	*Propionibacterium acidipropionici*	[57]
			*Propionibacterium shermanii*	[48]
Consuming succinate				
Firmicutes	*Clostridaceae*	*Clostridium*	*Clostridium kluyveri*	[58]
			*Clostridium C. difficile*	[49]
	*Acidaminococcaceae*	*Phascolarctobacterium*	*Phascolarctobacterium faecium*	[59]
		*Phascolarctobacterium*	*Phascolarctobacterium succinatutens*	[60]
	*Veillonellaceae*	*Dialister*	*Dialister propionicifaciens*	[60]
			*Dialister succinatiphilus*	[60]
		*Veillonella*	*Veillonella parvula*	[60]
	*Ruminococcaceae*	*Ruminococcus*	*Ruminococcus bromii*	[39]
Bacteroides	*Odoribacteraceae*	*Odoribacter*	*Odoribacter spp*.	[49]
	*Bacteroidaceae*	*Bacteroides*	*Bacteroides thetaiotaomicron*	[61]

## MULTIFACETED FUNCTIONS OF SUCCINATE AS A SIGNALING TRANSMITTER IN DISEASES

**Figure 4:**
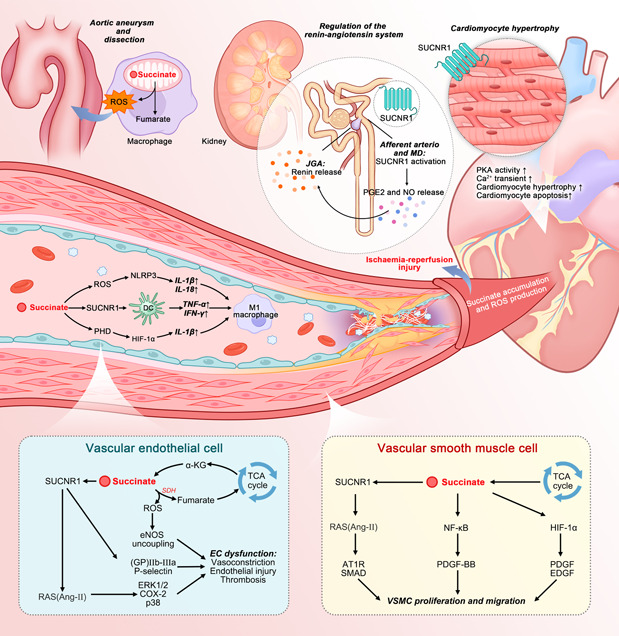
Effect of succinate on CVDs. Current research suggests the pleiotropic functions of succinate in vascular endothelial injury, VSMC growth and invasion, ischemia–reperfusion injury, macrophage polarization, aortic aneurysm and dissection, regulation of the renin‐angiotensin system, and cardiomyocyte hypertrophy, among others. Briefly, succinate can induce ROS in macrophages, promoting aortic aneurysm and dissection. It can also exacerbate endothelial cell dysfunction by upregulating ROS levels, which impairs the vasodilative effect of nitric oxide, activates RAS, and enhances thrombosis (shown in the blue rectangle at the bottom left). Accumulated succinate can stimulate SMC growth and invasion through RAS activation, HIF‐1α accumulation, and NF‐kB pathway promotion (shown in the yellow rectangle at the bottom right). In addition, succinate stimulates dendritic cells and macrophages to produce pro‐inflammatory cytokines, aggregating atherosclerosis. The interaction between succinate and SUCNR1 can disrupt the negative feedback loop of angiotensin II, contributing to hypertension and promoting cardiac hypertrophy via PKA pathway activation, Ca^2+^ transient, and cardiomyocyte apoptosis. Finally, accumulated succinate can drive ROS production at complex I, inducing ischemia‐reperfusion injury. Ang‐II, angiotensin II; AT1R, angiotensin II type‐1 receptor; COX‐2, cyclooxygenase‐2; DC, dendritic cell; EC, endothelial cell; EDGF, epidermal growth factor; eNOS, endothelial nitric oxide synthases; ERK1/2, extracellular signal‐regulated kinase 1/2; GP, glycoprotein; HIF‐1α, hypoxia‐inducible factor 1‐alpha; IFN, interferon; IL, interleukin; JGA, juxtaglomerular apparatus; MD, macula densa; NF‐kB, nuclear factor kappa beta; NLRP3, Nod‐like receptor 3; NO, nitrogen monoxide; PDGF‐BB, platelet‐derived growth factor‐BB; PGE2, prostaglandin E2; PHD, prolyl hydroxylase domain; PKA, protein kinase A; RAS, renin–angiotensin system;  ROS, reactive oxygen species; *SDH*, succinate dehydrogenase; SMAD, drosophila mothers against decapentaplegic protein; SUCNR1, succinate receptor 1; TCA, tricarboxylic acid; TNF, tumor necrosis factor; VSMC, vascular smooth muscle cell; α‐KG, alpha‐ketoglutarate.

Gut microbiota‐derived metabolites have garnered significant interest among researchers due to their pivotal regulatory functions. Succinate, a prominent signaling molecule, plays a role in numerous physiological activities and disease pathogenesis [62], leading to extensive investigations into its underlying mechanisms. With the continually advancing knowledge of gut microbiota, the role of succinate in CVDs has been increasingly recognized over the past decade. The present study examines the effects of succinate on CVDs and elucidates the associated mechanisms (Figure 4).

## AORTIC ANEURYSM AND AORTIC DISSECTION

Aortic aneurysm, often referred to as the “silent killer,” is characterized by the deterioration of the arterial wall and the dilation of the aorta, commonly accompanied by aortic dissection and acute aortic complications. The occurrence of aortic dissection can be attributed to several factors, including hypertension, advanced age, male gender, and smoking [63, 64]. AAD is a life‐threatening condition characterized by its sudden onset and high mortality rate. A significant number of aortic aneurysms occur without prior symptoms, leading to aortic rupture and subsequent sudden death. According to the Global Burden of Disease Study, the global death toll attributed to aortic aneurysms was 172,427 in 2019, representing an 82.1% increase compared to the figure in 1990 [65]. However, in the face of this highly fatal disease, there are currently limited effective therapies available to prevent or halt the progression of AAD [63, 66].

Remarkably, our research has made significant contributions to the management and treatment of AAD, introducing a completely novel paradigm. This study represents the first application of nontargeted metabolomics to characterize the metabolic landscape in AAD cases, leading to the identification of the crucial role of succinate in the disease [38]. In summary, the levels of succinate were found to be elevated in AAD cases compared to those in healthy individuals. This trend persisted when comparing them to cases of acute myocardial infarction (AMI) and pulmonary embolism (PE), indicating the potential use of succinate as a biomarker for AAD diagnosis and for distinguishing it from chest pain in AMI and PE patients. Moreover, the addition of succinate worsened AAD formation in mice, leading to increased mortality rate, higher AAD incidence, and enlarged aortic diameter, primarily through the excessive production of reactive oxygen species (ROS). We also provided evidence suggesting that the p38a–cyclic adenosine monophosphate‐responsive element‐binding protein 1–oxoglutarate dehydrogenase axis regulates succinate generation in macrophages and that p38a deficiency suppressed AAD development, further demonstrating the potential therapeutic effects of succinate in AAD. The significant value and potential of succinate in the future management of aortic aneurysm and aortic dissection are highly promising, underscoring the need for further research to fully elucidate the role of this metabolite in the disease.

## ATHEROSCLEROSIS

As a primary underlying pathology in various cardiovascular diseases, particularly coronary artery diseases, atherosclerosis initiates with the formation of lipid streaks on the vascular intima and progresses to the development of atheromatous plaques. The progression of atherosclerosis is primarily driven by inflammation and oxidation. Inflammatory cytokines associated with pro‐atherosclerosis processes trigger endothelial cell dysfunction, thereby initiating the advancement of atherosclerosi [67]. Following this, activated endothelial cells release growth factors and pro‐inflammatory chemokines, which lead to the transformation of vascular smooth muscle cells and promote the development of atherosclerotic lesions and fibrous plaques [68]. Activated endothelial cells play a critical role in recruiting inflammatory cells, such as macrophages and dendritic cells, which adhere to the endothelial cells and subsequently invade the intima. The interaction between these cells contributes to the progression of atherosclerotic damage and the rupture of plaques, potentially leading to myocardial infarction and mortality [69].

## EXCESS SUCCINATE ACCUMULATION IN ATHEROSCLEROSIS

A growing body of evidence has demonstrated that succinate levels are significantly increased in the context of hyperlipidemia, and excessive succinate plays a critical role in several pathological processes associated with atherosclerosis [70]. The upregulation of succinate in atherosclerosis can be attributed to high levels of fat, glucose, and lipopolysaccharide, which compromise the activity of succinate dehydrogenase and lead to the accumulation of succinate as a reaction substrate [71]. Furthermore, there is an increased conversion of glutamine to succinate through the “γ‐aminobutyric acid shunt” pathway [72], and more isocitrate is metabolized into succinate via the glyoxylate shunt pathway [73].

## PATHOGENESIS OF SUCCINATE IN DIFFERENT CELLS INVOLVED IN THE PROGRESSION OF ATHEROSCLEROSIS

The accumulation of succinate plays a role in the progression of atherosclerosis by influencing various cell types, including endothelial cells, smooth muscle cells, macrophages, and lymphocytes [74].

### Endothelial cells

Succinate has been shown to significantly exacerbate endothelial cell dysfunction [74]. One way it achieves this is by promoting the upregulation of ROS within mitochondria. This occurs through the translocation of cytosolic Drp1 to the outer mitochondrial membrane [74]. Additionally, succinate in its oxidized form is capable of increasing ROS production via a process called reverse electron transfer. This process impairs the vasodilatory effects of nitric oxide on the endothelium, thereby encouraging endothelial dysfunction [75]. A heightened level of ROS can also disrupt nitric oxide synthesis, further exacerbating endothelial dysfunction. Apart from its effect on ROS, succinate has also been observed to stimulate the renin‐angiotensin system (RAS) via its interaction with the succinate receptor 1 [SUCNR1, also known as G protein‐coupled receptor‐91 (GPR91)]. This interaction leads to an increased level of angiotensin II, promoting vasoconstriction and suppressing nitric oxide production, which in turn contributes to endothelial dysfunction [76, 77]. Moreover, an interplay between succinate and GPR91 on platelets augments the production of thromboxane A2, which in turn triggers platelet activation. This activation plays a crucial role in initiating and progressing atherosclerosis [77]. Platelet adhesion to the endothelium can damage endothelial cells, instigating a pro‐inflammatory response and potentially leading to thrombotic events. Another noteworthy metabolite is TMAO, which has been linked to increased platelet reactivity. This condition fosters thrombus formation through a mechanism that involves an elevated release of Ca^2+^ from intracellular stores, thereby associating TMAO with atherosclerosis [27, 78]. Given the impacts of both succinate and TMAO on atherosclerosis risk, a more thorough investigation of their combined effects is indeed warranted.

### Smooth muscle cells

The accumulation of succinate has been found to instigate the growth and invasion of smooth muscle cells (SMCs) into the intima, a phenomenon observed in atherosclerosis [79]. The pathological progression of SMCs can be attributed to three primary pathways. Firstly, succinate can stimulate the activation of the RAS, leading to the release of angiotensin II. This compound then spurs fibrosis, SMC growth, and hypertrophy through the SMAD pathways. Furthermore, angiotensin II can enhance the generation of pro‐inflammatory factors and growth factors, thereby prompting SMCs to transition from a contraction state to a synthetic state. Once transformed, SMCs produce pro‐inflammatory cytokines and components of the extracellular matrix (ECM), processes which collectively exacerbate atherosclerosis. The second pathway involves elevated succinate contributing to the accumulation and transcription of hypoxia‐induced factor‐1α (HIF‐1α) via the oxidation of Fe^2+^ [79]. The increase in HIF‐1α stimulates SMC growth by promoting various growth factors and mitochondrial division. The third pathway is characterized by succinate activating the nuclear factor kappa beta (NF‐κB) signaling pathway via agonists of NF‐κB ligands. This activation escalates the synthesis of cytoskeletal proteins in SMCs and drives their phenotypic differentiation, proliferation, and migration through the NF‐κB/mammalian target of rapamycin pathways.

### Immune cells

Succinate can also incite the polarization of pro‐inflammatory phenotype macrophages, thereby augmenting atherosclerosis [80]. Increased succinate levels lead to a rise in HIF‐1α, which subsequently triggers the production of interleukin (IL)−1β [81]. Additionally, succinate‐derived ROS and the interaction between succinate and GPR91 facilitate the generation of pro‐inflammatory factors such as IL‐1β, IL‐18, TNF‐α, and TNFβ, which induce the polarization of pro‐inflammatory phenotype macrophages [82]. Dendritic cells also participate in atherosclerosis pathogenesis [83]. Succinate can provoke these cells to produce pro‐inflammatory cytokines by activating GPR91, which is expressed on the surface of dendritic cells. The cytokines TNF‐α and IFN‐γ, produced by dendritic cells, can advance atherosclerosis and sustain pro‐inflammatory phenotype macrophages [84].

Recent evidence suggests that the role of lymphocytes, including B and T cells, is crucial to the pathogenesis of atherosclerosis [85, 86, 87]. B cells possess unique functions in response to injury, stress, and infection, including intercellular contact, cytokine generation, and antigen presentation. These cells are associated with local and systemic immunity, which promotes the progression of atherosclerosis [88]. Specifically, in the context of dyslipidemia, activated endothelial cells that cover atherosclerotic plaques enable various immunoglobulins produced by B cells to penetrate plaque areas, thereby performing distinct functions. In the late stages of plaque formation, tertiary lymphatic structures, such as the outer membrane of the artery develop, where plasma cells can also produce immunoglobulins in situ. Moreover, B cells are capable of producing a range of factors, including pro‐atherosclerotic TNF and anti‐atherosclerotic IL‐10, which further the progression of the disease [85]. In recent years, a variety of T‐cell subtypes have been identified as participants in atherosclerotic disease progression, playing diverse roles. These include pro‐inflammatory CD8 T cells, pluripotent CD4 T cells, and anti‐inflammatory regulatory T cells [86, 89]. The most recent findings indicate that several T cell peripheral immune checkpoints are compromised in the immune microenvironment within atherosclerotic plaques, which could be a key factor propelling the progression of atherosclerotic disease [90]. Over the past decades, mounting evidence has suggested a connection between immune cells and the pathogenic mechanisms of atherosclerosis. However, the relationship between succinate and lymphocytes in atherosclerosis remains unexplored. The potential correlation between succinate and atherosclerosis pathogenesis through the regulation of B and T cell functions presents an intriguing avenue for future research.

## POTENTIAL TREATMENT PERSPECTIVES OF SUCCINATE IN ATHEROSCLEROSIS

Therapeutic strategies targeting the succinate‐induced atherosclerosis pathway have been shown to offer cardiovascular protection. The inhibition of NF‐κB can mitigate the hydrolysis of matrix metalloproteinase‐9 and restrain the migration of smooth muscle cells, thereby offering protection against plaque rupture. Additionally, cinnamaldehyde, which is capable of suppressing the production of succinate‐induced HIF‐1α and IL‐1β, can also attenuate the inflammatory response induced by succinate in atherosclerosis [91]. These findings underscore the promising potential for the clinical translation of succinate‐based therapies in atherosclerotic CVDs.

## RENIN ANGIOTENSIN SYSTEM AND HYPERTENSION

Succinate has the ability to activate GPR91, also known as SUCNR1, which is typically found within the vascular lumen of the kidney, predominantly in the afferent arterioles and glomerular vasculature [92, 93]. Activation of GPR91 can augment the production of cyclooxygenase‐2 [94], culminating in the synthesis and secretion of prostaglandin E2. This then interacts with the EP2/EP4 receptors located in granular cells [94], which stimulates the production of renin by the granular cells in the juxtaglomerular apparatus. This interaction leads to dilation of the afferent arteriole. The association between succinate and GPR91 bypasses the negative feedback loop of angiotensin II, potentially leading to hypertension. Typically, the release of renin is controlled by the angiotensin II‐mediated negative feedback loop. The interaction of angiotensin II and its receptor inhibits the release of renin through the calcium protein kinase C pathway [95]. However, under hypertensive pathological conditions, the GPR91 signal and GPR91‐induced renin release contribute to the formation of angiotensin II, which can trigger the synthesis of (pro)renin in the tubule [96]. The communication between (pro)renin and its receptor, coupled with activated GPR91, enhances the phosphorylation of extracellular signal‐regulated kinase 1/2 (ERK1/2), aiding in the proliferation of tubular cells and tubulointerstitial fibrosis [97].

## CARDIAC HYPERTROPHY

Succinate has the capacity to induce cardiomyocyte hypertrophy by directly activating GPR91 in cardiomyocytes, a receptor broadly distributed throughout the body [98]. In the kidney, GPR91 is expressed within the renal vascular lumen, tubules, and Henle's loop [92, 93, 94]. In contrast, ventricular GPR91 is primarily located within the T tubules and sarcolemma membrane of cardiomyocytes within the heart [99]. The engagement of succinate and GPR91 in cardiomyocytes activates two independent intracellular signaling pathways leading to hypertrophy. One involves the stimulation of the MAP/ERK kinase, resulting in the phosphorylation of ERK1/2. The phosphorylated ERK1/2 within the nucleus can initiate gene transcription associated with cardiac hypertrophy. The second pathway involves the activation of phospholipase C, which produces diacylglycerol and inositol‐3‐phosphate. The binding of inositol 3,4,5‐triphosphate to its receptor promotes the release of Ca^2+^ into the cytosol. This release triggers calcium/calmodulin‐dependent protein kinase IIδ activation, which phosphorylates histone deacetylase 5, moving it to the nucleus and subsequently facilitating the transcription of hypertrophic genes [98, 100].

In addition to activating GPR91 in cardiomyocytes, succinate also contributes to cardiac hypertrophy by activating GPR91 in the kidney, which then activates the RAS and increases mean arterial blood pressure [101]. Studies in rodents have shown that losartan, a RAS antagonist, can mitigate the succinate‐induced increase in mean arterial blood pressure. However, it does not reverse succinate‐induced cardiac hypertrophy. This suggests that while succinate can activate RAS, this activation is merely one of several pathways through which succinate induces cardiac hypertrophy [99]. Further rodent experiments have shown that succinate‐induced cardiomyogenic hypertrophy is GPR91‐dependent; cardiac hypertrophy does not occur following exposure to succinate once GPR91 has been knocked out [99].

## REPERFUSION INJURY IMPAIRMENT

The heart plays a crucial role in circulating blood throughout the body and has a high dependence on oxygen. When the supply of oxygen becomes insufficient, as in the case of ischemia, myocardial metabolism can be profoundly impacted. This insufficiency in cardiac perfusion leads to a deficit in ATP and an accumulation of several metabolites, including lactate and succinate [102, 103, 104].

A considerable increase in succinate levels under ischemic conditions has been documented in various animal models, such as hypoxic rabbit papillary muscles [102] and isolated rat hearts [105]. This elevation is, therefore, a fundamental characteristic of ischemia and can serve as the electron source for ROS generation during reperfusion. Unlike normal conditions where succinate is produced via the citric acid cycle by the oxidation of fatty acid‐ and glucose‐derived carbon, during reperfusion injury, succinate is also synthesized through the mitochondrial reaction of amino acids [106]. The abnormal buildup of succinate under ischemia can primarily be attributed to two pathways. Firstly, due to the increased NADH/NAD^+^ ratio caused by ischemia, the normal conversion of α‐ketoglutarate into succinate via succinyl‐CoA is significantly impaired. This has been confirmed in animal models that exhibit a failure of α‐ketoglutarate to convert into succinyl‐CoA under ischemic and hypoxic conditions [107]. Secondly, the reverse action of complex II, also known as reverse electron transport, contributes to the production of ischemic succinate during ischemic reperfusion injury. This is where succinate receives an electron from the reduced coenzyme Q, enabling complex I to pump protons independent of oxygen [108]. These two pathways of ischemic succinate generation have been verified in both in vitro and in vivo animal experiments [109].

The accumulated ischemic succinate prompts the production of ROS at complex I during reperfusion. In addition to ROS production, succinate also induces an excessive release of intracellular calcium. Accumulated succinate can activate protein kinase A, leading to an increased release of intracellular calcium transients with higher peak height and frequency, thereby impairing cardiomyocyte contraction. Due to the enhanced activation of protein kinase A, the excessive intracellular calcium release, and ROS, cardiomyocyte apoptosis is significantly elevated [100].

Given the pivotal role of succinate in ischemic reperfusion injury, therapies targeting the succinate generation pathway present potential treatment options [110]. According to in vivo experiments, the administration of 5‐Aminoimidazole‐4‐carboxamide ribonucleotide and aminooxyacetate—which inhibit the conversion of α‐ketoglutarate to succinate and the acceptance of an electron from coenzyme Q by succinate, respectively—could reduce the production of ischemic succinate [109]. Moreover, dimethyl malonate, a complex II inhibitor, can reduce succinate levels and ROS generation during ischemia, subsequently reducing infarct size. The infusion of dimethyl malonate in isolated rat hearts has also been shown to protect against ischemic reperfusion injury [111, 112]. These findings indicate potential therapeutic applications for counteracting the detrimental effects of succinate overproduction, though further clinical evaluations are warranted. In summary, the effects of succinate on CVDs are displayed in Figure 4.

## CONCLUSIONS AND PERSPECTIVES

Succinate plays a role not only in the physiological TCA cycle but also drives numerous pathophysiological processes, such as the activation of RAS, overproduction of ROS, mediation of pro‐inflammatory macrophages, interaction with GPR91, and involvement in energy metabolism. Both the TCA biosynthesis pathway and gut microbiota metabolism participate in the production and regulation of serum succinate levels. The multifaceted activities of succinate contribute to the onset and progression of various cardiovascular diseases, including amplified aortic aneurysm, aortic dissection, atherosclerosis, RAS activity, hypertension, cardiac hypertrophy, and impairment due to reperfusion injury. Antagonists of succinate signaling pathways can effectively mitigate cardiovascular diseases and offer protection against succinate‐induced ROS overload and inflammatory responses, thereby emerging as promising therapeutic targets. Given that gut microbiota‐derived metabolites are important in many diseases, treatments targeting gut microbiota have garnered significant interest.

Although fecal microbiota transplantation was initially proposed as a treatment strategy for various diseases, the results have been less satisfactory due to population heterogeneity, highlighting the need for more precise treatment targets within gut microbiota. Presently, several gut microbiota‐related metabolic pathways are considered potential therapeutic targets to combat cardiovascular diseases. Concentrating on gut microbial enzymes to diminish the production of “adverse” metabolites or to promote beneficial microbial biosynthesis pathways is deemed to be a promising therapeutic approach for disease control. Treatments focusing on succinate and its associated metabolic pathway present a novel and precise therapeutic potential, considering succinate's role in the initiation and progression of several cardiovascular diseases, as well as signal transmission. Capsule forms of succinate pathway inhibitors could enhance clinical administration compared to traditional fecal microbiota transplantation.

In spite of the expanding literature on succinate and the gut microbiome, many questions remain unanswered. For instance, prior research has illuminated the advantageous effects of succinate, particularly its promotion of thermogenesis in brown and beige adipose tissue as a counter to metabolic diseases [113]. Studies have demonstrated that succinate administration can enhance glucose and insulin tolerance in mice, reflecting its positive impact on glycemic control [43]. Moreover, succinate's role in ameliorating intestinal inflammation has been highlighted [114]. This raises inquiries regarding the comprehensive understanding of this metabolite. The exploration of whether these conflicting effects result from dosage effects, compensatory effects, or organ specificity of succinate is warranted for further scrutiny. At present, the regulation of succinate production in the gut remains uncertain, including whether its effects on the microbiome fluctuate based on the type of dietary fiber consumed. Both gut microbiota and human mitochondria contribute to plasma succinate levels, yet the proportion of succinate originating from human cells and from gut microbes, along with their interaction with pathogenesis, remains to be established. Additionally, there is a need for more research to comprehend the long‐term effects of succinate supplementation on host metabolism and immune function. It is crucial to acknowledge that a significant portion of the research on succinate and the gut microbiome has been carried out in animal models. Hence, further research is necessary to verify these findings in human studies.

The burgeoning evidence surrounding the role of succinate in the gut microbiome carries significant implications for our comprehension of host‐microbiome interactions and the development of new treatments for CVDs. Further investigations are essential for thoroughly understanding the underlying mechanisms and applying these findings in clinical practice.

## AUTHOR CONTRIBUTIONS

Jing Xu, Yicheng Yang, and Xin Li wrote the manuscript. Jing Xu, Yicheng Yang, and Shusi Ding revised the manuscript. Lemin Zheng, Changming Xiong, and Yuejin Yang supervised this project. All authors have read the final manuscript and approved it for publication.

## CONFLICT OF INTEREST STATEMENT

The authors declare no conflict of interest.

## Data Availability

This manuscript does not generate any code or data.
